# A red flag: an experimental analysis of the function of pink colouration in males of *Calopteryx haemorrhoidalis asturica* (Odonata: Calopterygidae)

**DOI:** 10.1098/rsos.240144

**Published:** 2024-06-12

**Authors:** Xin Yu, Anais Rivas-Torres, Adolfo Cordero-Rivera

**Affiliations:** ^1^ Universidade de Vigo, ECOEVO Lab, E. E. Forestal, Campus Universitario, Pontevedra 36005, Spain; ^2^ College of Life Sciences, Chongqing Normal University, Chongqing 401331, People's Republic of China

**Keywords:** sexual selection, female choice, courtship, post-copulatory behaviour, fat content, muscle mass

## Abstract

Males display phenotypic characteristics that may be associated with their quality, allowing non-random mating and post-copulatory female choice. In the damselfly *Calopteryx haemorrhoidalis asturica*, males have a conspicuous pink colouration in the underside of abdominal segments 8–10, which they exhibit during pre- and post-copulatory courtship. We hypothesized that this colouration functions to increase male mating success and/or to elicit females to oviposit. We estimated mating and oviposition success of 27 males, and on the following day, treated males had their segments 8–10 painted black and control males the seventh segment. We recorded the number of male–male fights and courtships, whether the courtship ended in copulation, and whether the female remained in the territory and laid eggs. Our results indicate that the mating success of male *C. h. asturica* was not significantly affected by the removal of the pink colouration of the abdominal tip, but this colouration clearly affected their success in enticing females to oviposit. Courtship frequency, fat content and muscle mass were positively correlated to male mating rate, and the number of aggressive encounters was negatively correlated. Our study yields experimental evidence for the function of pink colouration of male *C. h. asturica*, in the context of post-copulatory sexual selection.

## 1. Introduction

Many sexually selected characters are used in pre-copulatory interactions, where typically males show ‘badges’ of quality, and females use these characters in their reproductive decisions, thereby producing mate choice based on these traits [1]. Sexual selection is, therefore, the conceptual framework behind the study of most reproductive behaviours and has a competitive nature, which, nevertheless, does not exclude cooperation [2]. At the heart of pre-copulatory sexual selection is the concept of mate choice: a preference that arises from phenotypic attributes in one sex that lead to non-random mating by the other [3]. In most species, males show phenotypic unique characteristics that are favoured by female choice, and these can be arbitrary, i.e. Fisherian selection [4], or related to male quality, i.e. good genes hypothesis [5]. However, distinguishing between these alternatives is not an easy task [6]. It is also relevant to keep in mind that genetic compatibility between females and males might affect the outcome of sexual selection [7].

The relationship between sexually selected characters and male mating success has been studied in many species, particularly in lek mating systems, where apparently these characters are honest indicators of male quality [8]. In the case of Odonata (damselflies and dragonflies), many species show territorial behaviour, or at least aggressive, with males defending particular areas with plants to be used as oviposition substrate, a fact common in the family Calopterygidae (reviewed by [9]). Males can also behave in a similar way to lekking systems, concentrating in areas that do not have any resource of interest to females [10,11]. In odonates, large body size has been found to be positively correlated with territoriality [12]. In territorial species, males commonly show pigmented wings which are attractive to females [13,14], or other secondary sexual characteristics that may correlate with mating success, like for instance head colour pattern in *Tigriagrion aurantinigrum* [15] or the enlarged tibiae of Chlorocyphidae [16,17] or Platycnemididae [18] (but see [19]).

**Figure 1.**
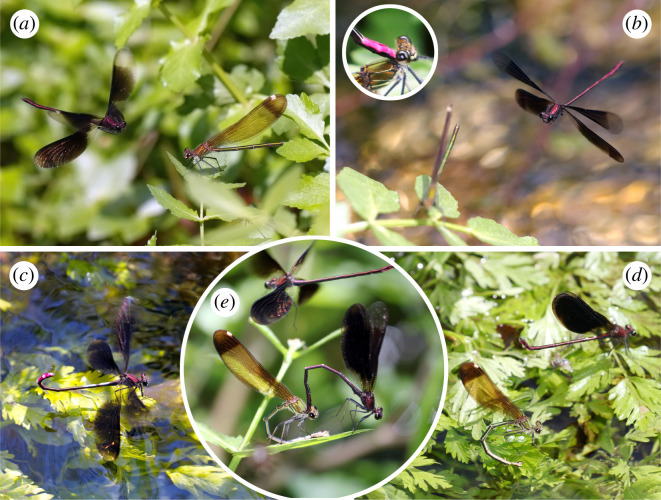
Copulation behaviour of *Calopteryx haemorrhoidalis asturica*. (*a*,*b*) Males courting females with the courtship arc, with the abdomen tip slightly upwards. Note that the pink colouration (insert in *b*) is not visible to females in this position. (*c*) A male performing the cross display over the surface of the water, with the abdominal tip bent to clearly show the pink colouration. A second example of the courtship arc, in this case to an ovipositing female, is shown in (*d*). A black-painted male with a female in tandem is presented in (*e*). Note that the pink colouration starts to reappear after the treatment. Photos by A.C.-R.

*Calopteryx haemorrhoidalis* is a common territorial damselfly found in the Mediterranean region of Europe [20]. The behaviour of the subspecies *Calopteryx haemorrhoidalis asturica* [21] has been previously studied by observational and manipulative experiments. Córdoba-Aguilar [22] described the reproductive behaviour of territorial and non-territorial males and showed that territorial males court females arriving at their territories by a cross display [23] and/or a courtship arc [24]. Males fly around and in front of the perched female (courtship arc; figure 1*a*,*b*), using mainly their forewings to sustain flight, and may land on the water or a substrate (cross display; figure 1*c*,*d*). In both cases, males bend up their last four abdominal segments, showing the conspicuous pink colouration present in the ventral surface of segments 8–10 (figure 1*b*,*c*) [25]. The function of this pink colouration and the associated display has not been studied. We hypothesized that the pink colouration is an honest signal of territorial male quality, which is used by females in their reproductive decisions, implying that the absence of this colouration should reduce male mating success.

On the other hand, *C. h. asturica* males have their wings heavily pigmented, but in some populations, there is a large variation in the extent of this pigmentation (see fig. 2*b* in [9]), sometimes as a response to the presence of congeneric species (e.g. [26]), and experiments have shown that males of *C. h. asturica* with a higher proportion of their wings pigmented have higher survival, territorial success and mating success [27]. Considering this information, we predicted that males with higher pigmentation proportion would be more successful in mating.

**Figure 2.**
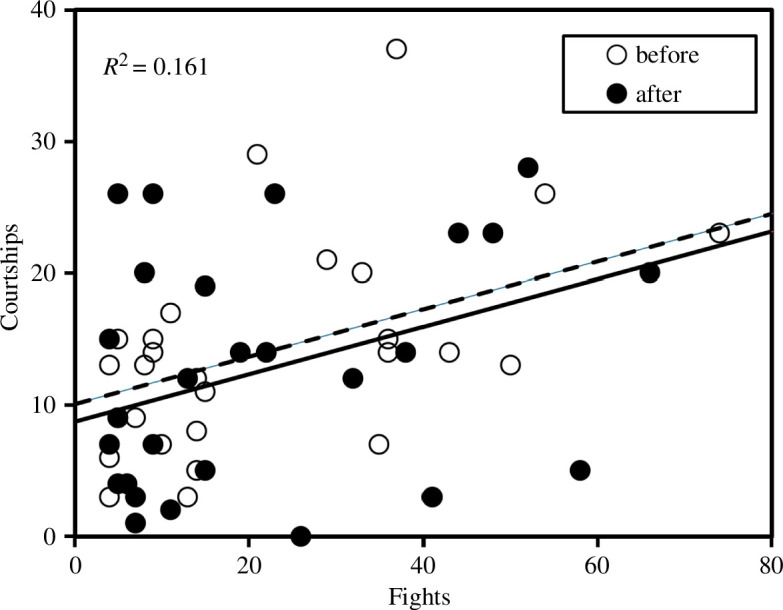
The relationship between aggressivity (number of fights) and courtship activity (number of courtships to females) in males of *C. h. asturica*. Each male contributes with two data points in this plot, before (dotted line) and after the treatment (continuous line).

Finally, calopterygid males are well known for their aggressive territorial disputes, which normally end after a few seconds, with the resident male winning most of the disputes (e.g. [28]), but escalated fights are won by the males with higher energy (fat) reserves [29,30]. Therefore, the amount of fat and muscle mass that males possess may predict their ability in territorial contests and, indirectly, in mating success [31].

Here, we present an experimental analysis of the behaviour of males of *C. h. asturica*, where we manipulated (obscured) the pink abdominal colouration, and estimated their short-term reproductive success, wing pigmentation, body size, muscle mass and fat reserves.

## 2. Methods

### 2.1. Study species and site

We studied a population of *C. h. asturica* living in the stream Río da Chanca in Meaño (Pontevedra, northwestern Spain; coordinates 42.436227° N, 8.795318° E, 28 m altitude), on 23 days between 14 July and 24 August 2020, with an average of 3.7 h of observation per day, and a total of 84 h of fieldwork. Observations started around 13.00 (official time; solar time is 2 h earlier) and ended 4–5 h later, when activity was clearly reduced. Using a datalogger (Tinytag, Gemini dataloggers), we recorded air temperature in the shade of an *Alnus glutinosa* tree every 5 min. *Calopteryx h. asturica* was the dominant damselfly species in the river but coexisted with a small population of *Calopteryx virgo* (Linnaeus, 1758), and males sometimes engaged in interspecific fighting. Other odonate species common in the river include *Boyeria irene* (Fonscolombe, 1838), which predated *C. h*. *asturica* [32], *Onychogomphus uncatus* (Charpentier, 1840) and *Cordulegaster boltonii* (Donovan, 1807).

### 2.2. Experimental design

Males of *C. haemorrhoidalis* show intense pink colouration on the underside of the last abdominal segments (figure 1*b*), and this colour seems to be displayed in females during pre- and post-copulatory courtship [22,33]. In our experience, the size of the pink colour is similar among individuals, covering segments 8–10 and the underside of the paraprocts, and achieving the highest intensity in fully mature males (for a picture, see [25]). To test if pink colouration affects male copulation success, we randomly applied a treatment to some males, which consisted of fully covering the pink colour with black paint, using a permanent marker (Fila Group). Control males were captured and handled in the same way but were painted on segment 7, hence leaving the pink colouration intact, but being in contact with the paint, thus controlling the effect of the manipulation procedure (see electronic supplementary material, figure S1, for examples of the treatment). All males were marked with a unique combination of white dots and lines on the dorsum of the abdominal segments 2–4, using a paint marker (MP Company) to allow easy identification. We refrained from marking the wings to avoid any possible interference of wing marks on male courtship success [14]. The age of males (immature, just mature and mature or old) was estimated by their wing flexibility [30]. All but two males included in the experiment were mature; the remaining two were just mature. We avoided including old males in the experiment because their mating success might be different [34].

Marking was done early in the morning, by selecting daily up to 10 males that showed territorial behaviour. These males were site-attached and attacked rivals, defending a territory on the water. After marking, males were released. If the male returned to the previous territory, we started a focal observation, which lasted an average of 3.31 ± 0.13 h, and recorded the number of fights with other males, the number of courtships, whether the courtship ended in copulation, and whether the female remained in the territory and laid eggs. When possible, we timed copulations to the nearest second and recorded the time of the start of stage II to estimate the duration of stages I and II. In damselflies, stage I is the longest and most variable phase of copulation, both intra- and interspecifically and its main function is to allow the male to remove sperm from the previous mates (see [35], but also to guard and court the female [36]). Stage II is less variable in duration and its function is to inseminate the female.

The day after the first focal observations, we performed the manipulation, again early in the morning. Focal males (those observed the previous day) were captured, and sequentially assigned to treatment or control, to have a similar number in each group, but randomly in relation to their previous observations. Using a permanent pen, we black-covered the pink colour of segments 8–10 in treated males and the segment 7 (which does not have pink colour) in control males. We recorded again their mating success. The time between the first focal observation and the second was 1 day, except for one treated male (3 days) and one control male (2 days). The treatment was effective in masking the pink colouration, at least for a human observer, but in four cases, the pink colouration started to resurface at the end of the observations (see figure 1*e*). After this manipulation, focal males were observed for an average time of 3.39 ± 0.10 h.

At the end of the observations, focal males were captured and transported to the laboratory. Males were killed by freezing, and their wings were mounted on a plastic sheet and scanned to estimate wing length (left wing, except if damaged), wing area and the pigmented area of the wing. The thorax was dissected, and legs removed, and immediately weighed to the nearest 0.1 mg, using an electrobalance (Denver XE Series Model 50). Afterwards, the thorax was dried at 65°C for 24 h and weighed again to estimate dry weight. Dry samples were submerged in chloroform for 8 h to extract fats and weighed again to estimate fat content [37], which is known to be a source of energy for odonates [29]. Finally, the thorax was treated with NaOH for 24 h to eliminate muscles [38], air-dried for 1 h, maintained for further 1 h in a dryer and weighed again. With this procedure, we were able to estimate the thoracic muscle mass of each male.

The experiment was designed to obtain paired data of each focal male, i.e. the mating success before the manipulation was compared with the mating success of the same male after the manipulation. Unfortunately, it was not possible to remove the black paint over the pink colouration, and therefore all males were older in the second focal period (1 day in all but two cases, see above). However, any temporal/ageing effect should be present on the control males as well, and the comparison between both dates should reveal the effect of the manipulation.

### 2.3. Statistical analyses

To test the effect of the treatment on mating success, we used a generalized linear mixed model (GLMM), using mating rate (number of matings per hour of observation) as the response variate, and normal errors. The model included as fixed terms Treatment*Time, where time refers to before/after the manipulation, the number of courtships, the number of fights, hindwing length (as an estimate of body size), fat reserves, muscle mass and the proportion of dark colouration of forewing and hindwing. We entered male/time as a random term to account for the effect of the repeated observations of the same male (before/after the manipulation). If the treatment changes mating success of males, then the interaction Treatment*Time should be significant, since only the mating success of treated males should be reduced, but the control males should have a similar success before and after the manipulation. A similar model was built to analyse the number of ovipositions obtained by the focal males, but in this case, with a Poisson distribution. The dispersion parameter was estimated and used to correct s.e. and statistical tests, as indicated in the electronic supplementary material tables. We analyzed the relationship between behavioural variables using linear models, as described in §3.

Means are presented with their standard errors and sample size in parentheses. Analyses were done with GenStat 23rd edition and xlStat 2021.

## 3. Results

### 3.1. Mating activity and copulatory behaviour

We started observations at around 13.00, but most matings took place in the afternoon (78.5% between 14.30 and 17.00), when temperatures were higher. Territorial males patrolled an area of 1–2 m^2^, making frequent interactions with their neighbours. When females appeared, males started courtship, using the courtship arc (figure 1*a*,*b*) before copulation, and the cross display (figure 1*c*,*d*), sometimes before but always after copulation. Males that were more active fighting were also more active courting females, as both behaviours were positively correlated (ANCOVA, *t*_51_ = 3.062, *p* = 0.004), but their behaviour before/after the treatment was similar (ANCOVA, *t*_2_ = −0.633, *p* = 0.530; figure 2).

**Figure 3.**
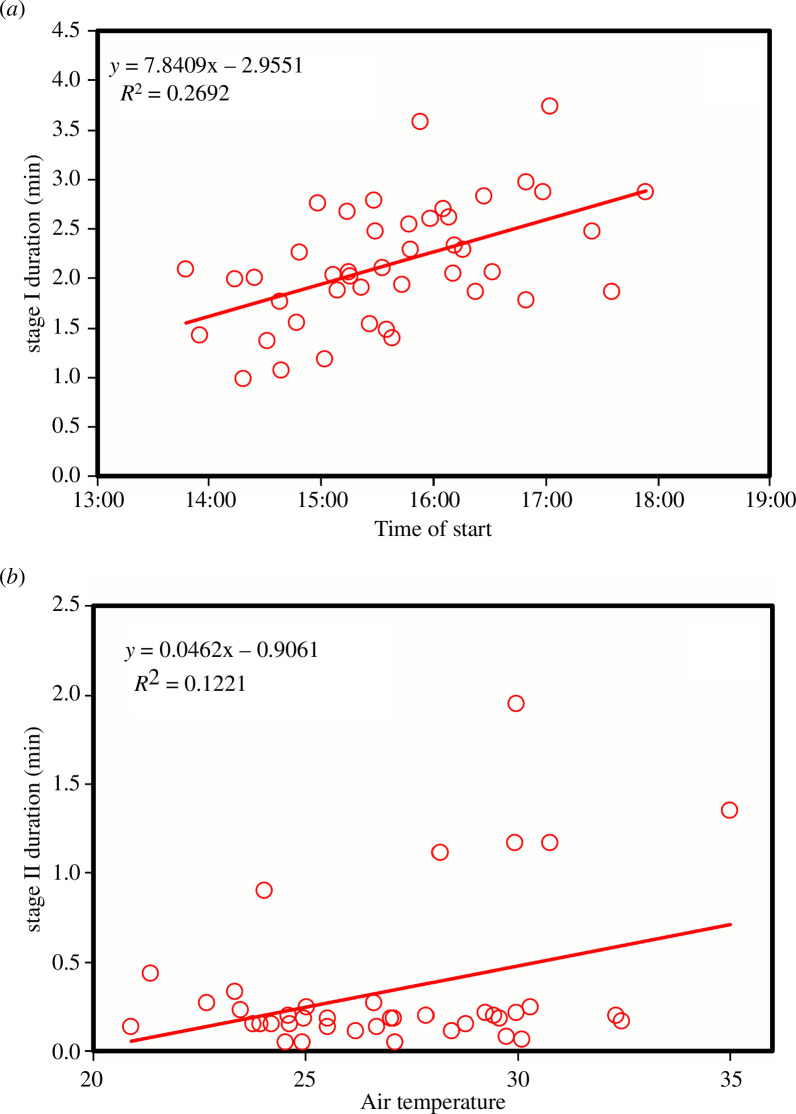
The relationship between (*a*) the duration of stage I of copulation and the time of start of copulation and (*b*) stage II duration and temperature in males of *C. h. asturica*.

We analysed the duration of copulations and their stages, including observations of 27 focal males, four males that did not appear on the second day, and five unmarked males. The average duration of copulations was 2.38 ± 0.10 (73) min, with stage I lasting 2.13 ± 0.09 (47) and stage II 0.35 ± 0.06 (47) min. Using a linear mixed model (LMM), we tested the effect of time of day and air temperature on copulation duration (Box–Cox transformed to normalize it), including male identity as the random term. The results indicate that these factors did not significantly affect copulation duration (time of start, *F*_1,62.9_ = 0.06, *p* = 0.810; air temperature, *F*_1,63.9_ = 2.55, *p* = 0.115). The duration of stages I and II could not be normalized using Box–Cox transformations. However, a LMM analysis as above suggests that stage I increases its duration with time of day (*F*_1,27.9_ = 6.89, *p* = 0.014; figure 3*a*) but is not affected by air temperature (*F*_1,35.5_ = 0.00, *p* = 0.962). In the case of stage II, the time of start was not significant (*F*_1,17.3_ = 1.51, *p* = 0.235), but there was a tendency to increase its duration with temperature (*F*_1,19.6_ = 3.02, *p* = 0.098; figure 3*b*).

### 3.2. The effect of pink colouration

We were able to mark and observe the behaviour of 27 focal males on both days, which were involved in a total of 60 matings, with a mean number of 2.6 ± 3.7 (23) matings observed per day. The average mating rate (matings per hour) was 0.34 ± 0.04 (27).

**Figure 4.**
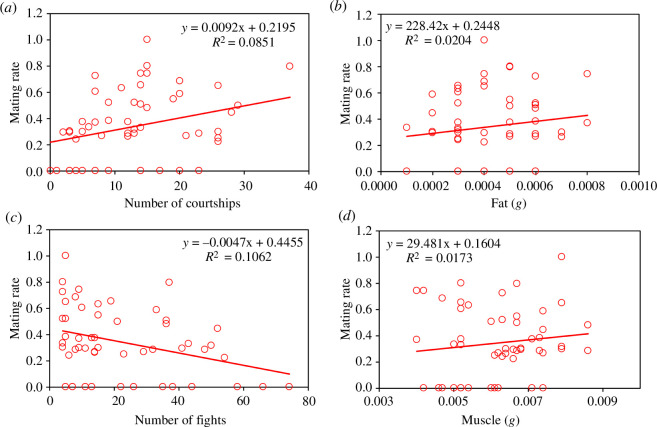
The effect of phenotypic variables on the mating rate in males of *C. h. asturica*. The mating rate increased with the courtship activity (*a*), and was positively related to fat reserves (*c*), decreased with fighting (*b*) and increased with muscle mass (*d*). However, note that the proportion of variance explained by any single variable is low, suggesting that other factors are affecting the mating rate.

Control males had a mean mating rate of 0.47 ± 0.08 (13) matings per hour before the treatment and 0.33 ± 0.08 (13) after the application of black paint to their seventh abdominal segment. Treated males had a mating rate of 0.32 ± 0.06 (14) before and 0.26 ± 0.06 (14) after the application of black paint to their segments 8–10. Therefore, both groups of males reduced their mating rate after the treatment. However, the GLMM analysis indicates that the treatment did not have a significant effect on the mating rate (*F*_1,42.1_ = 2.22, *p* = 0.144), time was close to significance (*F*_1,42.0_ = 3.45, *p* = 0.070), but the interaction Treatment*Time was not significant (*F*_2,42.0_ = 0.52, *p* = 0.477), indicating that the pink colour was not relevant in mating success. Nevertheless, four phenotypic variables were significantly related to mating rate: the number of courtships was positively correlated with the mating rate (*F*_1,42.4_ = 15.58, *p* < 0.001; figure 4*a*), the number of fights was negatively correlated (*F*_1,43.0_ = 11.29, *p* = 0.002; figure 4*b*), fat reserves were positively correlated (*F*_1,42.2_ = 4.19, *p* = 0.045; figure 4*c*), as well as muscle mass (*F*_1,42.1_ = 4.51, *p* = 0.040; figure 4*d*). Hindwing length and the proportion of pigmented area of the forewing and hindwing did not have a significant effect (the full analysis is presented in electronic supplementary material, table S1).

**Figure 5.**
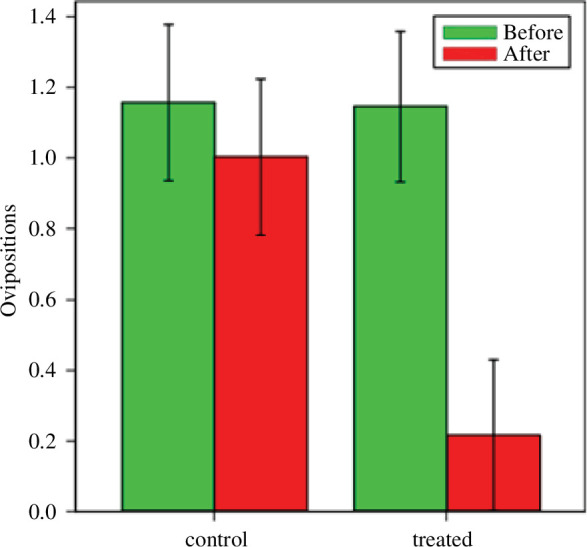
The mean number (±s.e.) of females that oviposited in the territories of focal males before and after the treatment for control males (painted on the seventh abdominal segment) and treated males (painted to cover pink colouration on segments 8–10). The interaction term is significant (*F*_2,41.7_ = 6.56, *p* = 0.014).

**Figure 6.**
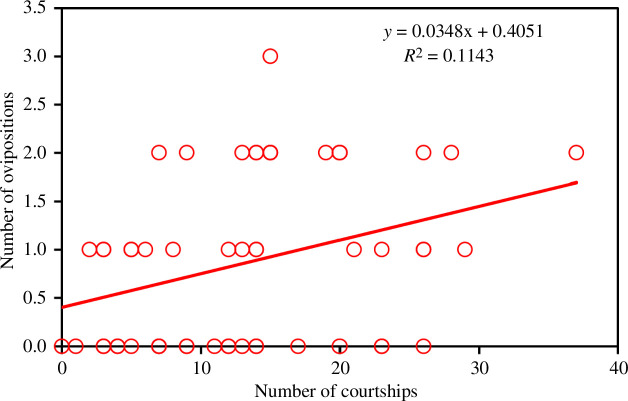
The relationship between the courtship intensity and the number of ovipositions obtained by male *C. h. asturica*.

The analysis of the number of females that remained laying eggs in the territories of the focal males was done with a GLMM with Poisson errors, with the same structure as for the mating rate. In this case, treated males had clearly a lower success, and most females did not lay eggs in their territory after the manipulation (figure 5). This analysis indicates that treatment did not have a significant effect on the oviposition success of males (*F*_1,41.3_ = 0.67, *p* = 0.418), time was close to significance (*F*_1,41.2_ = 3.13, *p* = 0.084), and the interaction Treatment*Time was significant (*F*_2,41.7_ = 6.56, *p* = 0.014), indicating that the pink colour was very relevant to elicit oviposition. In this case, the only phenotypic variable significantly related to oviposition success was the number of courtships, which was positively correlated (*F*_1,42.1_ = 6.45, *p* = 0.015; figure 6). The rest of the variables did not have a significant effect (the full analysis is presented in electronic supplementary material, table S2).

## 4. Discussion

Our results indicate that the mating success of male *C. h. asturica* was not significantly affected by the removal of the pink colouration of the abdominal tip, but this colouration clearly affected their success in enticing females to oviposit. Oviposition in *C. haemorrhoidalis* is made by the female alone, with non-contact guarding by the male, and therefore the female can easily abandon the territory where she has mated and decide to lay eggs in another territory. From the first day of observation (before the manipulation) to the second day (after being black painted on segment 7 or segments 8–10), there was a tendency to a decrease in mating rates, but this affected in a similar way to control and experimental males. However, the treatment clearly reduced the number of females that decided to lay eggs in the territory of treated males compared with control males (figure 5). Our general results are in agreement with previous research that indicates that courtship persistence and fat and muscle mass are positively related to mating success in male *Calopteryx*.

### 4.1. Copulatory behaviour

Calopterygids are characterized by short copulations, ranging from 1 to 5 min, and most of the time is dedicated to sperm removal (reviewed by [9]). Córdoba-Aguilar [22] indicates that copulation lasted an average of 2.8 min in a population of *C. h. asturica*, not far from our field site, stage I lasting 1.7 min and stage II 1.8 min. Our results are similar in relation to copulation duration (average of 2.4 min), but clearly divergent in relation to stage II duration, which in our experiments rarely lasted over 30 s (mean of 0.35 min). In fact, only 5 (10.6%, *n* = 47) matings showed stage II durations between 1 and 2 min (see figure 3*b*). In a population of the subspecies *Calopteryx haemorrhoidalis* in Italy, the duration of copulation was on average of only 1.6 min, with stage I lasting 1.4 min and stage II 0.3 min [35], values that are similar to our observations in the proportion of time dedicated to stage II (insemination). The difference with the observations of Córdoba-Aguilar [22] might be owing to the use of different criteria to define the start of stage II or inter-population differences.

Although damselflies are ectothermic animals, we found no effect of air temperature on mating duration, nor an effect of time of day on the whole copulation, but a positive effect on stage I duration (figure 3*a*). In species with very long copulations, like those of the genera *Ischnura*, *Enallagma* or *Ceriagrion*, mating has a guarding function, and therefore time of day has a negative effect on copulation duration [36,39,40], but in *Calopteryx*, copulation is brief and has no guarding function. The increase in stage I duration, which is used to remove rivals’ sperm from the female genitalia [35], could be explained if over the day females were more likely to be mated, and therefore males might need more time for sperm removal. However, in male *C. h. asturica*, the sperm displacement ability is related only to aedeagal width and has no relation to copulation duration or the number of stage I movements [41,42].

### 4.2. The function of pink colouration

We have found that male *C. h. asturica* show their pink colour during courtship, bending up their abdomen, but, in most cases, males face the female, and the abdomen is only slightly raised (figure 1*a*,*b*), making it unlikely that the female can see this colour during the courtship arc. However, males show this colouration to females by landing on the water or perching on floating plants, in the so-called cross-display [22] (figure 1*c*,*d*), which is sometimes also performed before copulation. The description of courtship in *C. haemorrhoidalis* by Heymer [33], which is based on the nominal subspecies, also indicates that males show the pink colouration to females only when they perch on the water, and therefore only at that moment can females evaluate male abdominal colouration. Therefore, our results corroborate that the pink colouration is not useful to obtain matings, and probably has evolved in the context of postcopulatory sexual selection, to elicit females to lay eggs in the territory of their mate. In fact, sometimes females accept to mate with males of another species of *Calopteryx* (one mating between male *Calopteryx virgo* and female *C. haemorrhoidalis* was observed during our fieldwork), which has a completely different colour in the abdominal tip, indicating that this colour is of little relevance during precopulatory courtship. These matings may produce hybrid offspring [25].

The pink colour apparently acts as an attractant to the best oviposition sites. Interestingly, females of another *Calopteryx* species frequently oviposit in the territory of a male without having mated with the territorial male, apparently deciding to use the sperm of sneaky males [43]. Therefore, the cross-display is very important to ‘convince’ the female to lay eggs, and in that way minimizing the possibility that the female decides to oviposit in another territory, and, probably, mating again. A similar display is performed by male *Calopteryx xanthostoma*, which have their last segments coloured in light yellow, male *Calopteryx virgo*, whose abdomen tip is light pink, and *Calopteryx splendens*, coloured whitish [33]. This behaviour seems part of the courtship of many calopterygids. For instance, males of the North American *Calopteryx aequabilis* court females with a cross display, while bending sharply upwards their 9–10 abdominal segments, always facing the female and almost touching the water with their hindwings [24,44]. Males of *Calopteryx maculata* do a similar display, showing the whitish colouration of the ventral side of the 9–10 abdominal segments [44,45]. Interestingly in *Calopteryx amata* and *Calopteryx atrata* the abdomen is held straight not showing the ventral colouration [44,46]. Finally, in *Neurobasis chinensis*, another member of the Calopterygidae, males also bend their last abdominal segments, showing their whitish colouration to females during courtship (the ‘tail lights’ of [47]), and similar behaviour is performed by *Matrona basilaris* (X.Y., personal observations). In several *Calopteryx* species, the male attempts to face the female at all times; thereby, the colouration of the abdomen tip is not visible to females during the precopulatory courtship arc [44,46].

In relation to phenotypic variables, our results point to a positive effect of male courtship intensity in mating (figure 4*a*) and oviposition induction (figure 6). Therefore, insistent males are more likely to obtain matings and to elicit females to lay eggs. Courtship behaviour has been found to be repeatable in *Calopteryx* males, indicating the existence of different personalities in field populations of these insects [48]. More active males were not only dedicating more time to court females but also more time to fight with their neighbours. However, very aggressive males were likely losing mating opportunities (figure 4*b*), perhaps because their courtships were frequently interrupted by their neighbours.

Since the pioneering experiments of Marden [29,31], it is known that fat reserves are a critical factor in territorial contests of male *Calopteryx*, which allow males to defend their territory for more time, and hence increase their mating success. Our results corroborate the relevance of fat reserves also in *C. haemorrhoidalis* (figure 4*c*), adding more support to the idea that fat reserves are important in calopterygids [30,49].

In territorial species, large body size is commonly positively correlated with mating success (e.g. [50], and in general, territorial species are larger [12]). However, we did not find a positive effect of size on mating rate in our experiment. In this species, larger males defend their territories for longer [27], and this is the main reason for their higher reproductive success because the daily mating rate is low. Therefore, given that our experiment only measured the mating rate on two consecutive days, the effect of size could be less relevant.

The administration of an analogue of the juvenile hormone to male *Calopteryx* has been found to increase aggressivity [51], reduce fat reserves and increase wing pigmentation [52]. These intricated effects suggest that the whole behavioural repertory of male *Calopteryx* is under the effect of sexual selection, and multiple collateral effects are expected. In fact, fat reserves are positively correlated with wing pigmentation in *Calopteryx maculata*, providing a cue to females to evaluate male condition [53] (but see [54]). Wing pigmentation was, nevertheless, not related to the mating rate in our experiment, in clear contrast with previous experiments with the same species, which found that more pigmented males obtained more matings and survived for longer, also maintaining their territories for more days [27]. One possible explanation is the fact that in our population wing pigmentation showed little variation, particularly on the hindwing (mean proportion of hindwing pigmentation: 0.87, range 0.82–0.90), which is the main target of sexual selection in calopterygids [55]. The evolution of hindwing shape in males is accelerated by sexual selection on pre-copulatory displays [56], and several damselflies with pigmented wings and territorial behaviour show divergent evolution of fore- and hindwings [57,58]. So, perhaps the wing shape is more relevant than pigmentation in this species. On the other hand, in *C. haemorrhoidalis*, the melanin is localized in the procuticle of the wings and is not associated with the lipids of the epicuticle [59], suggesting that the mechanism above mentioned for *C. maculata*, which relates fat reserved and wing pigmentation, might not be generalizable to other species. In fact, another study did not find a relation between pigmentation and fat reserves in *C. maculata* [54].

Finally, we found a positive correlation between muscle mass and mating rate (figure 4*d*). This is logical considering that flight manoeuvrability is crucial for successful courtship in the intricated vegetation of the streams inhabited by *C. haemorrhoidalis*. In fact, males of *Calopteryx atrata* allocate a high proportion of resources to develop thoracic muscle mass increasing about 2.4 times over maturation [60]. In *Hetaerina* damselflies, thoracic muscle mass is also a target of sexual selection [61]. The relevance of muscle mass has also been found in other families of territorial damselflies, like *Chalcolestes viridis*, where males with higher flight muscle mass were more likely to mate [62,63].

In conclusion, our study yields experimental evidence for the function of pink colouration of male *C. haemorrhoidalis*, in the context of post-copulatory sexual selection. Further experiments, for instance using dummy males with the pink colouration exposed, could be used to further test the function of this conspicuous colouration, and investigate if this pigmentation is costly, which is expected given the relevance of this trait for male reproductive success.

## Data Availability

All data are provided in the electronic supplementary material [64].
